# Identification of Dysregulated Complement Activation Pathways Driven by N-Glycosylation Alterations in T2D Patients

**DOI:** 10.3389/fchem.2021.677621

**Published:** 2021-06-11

**Authors:** Yang Zhao, Man Wang, Bo Meng, Ying Gao, Zhichao Xue, Minjun He, You Jiang, Xinhua Dai, Dan Yan, Xiang Fang

**Affiliations:** ^1^ Center for Advanced Measurement Science, National Institute of Metrology, Beijing, China; ^2^ College of Pharmacy, Chengdu University of Traditional Chinese Medicine, Chengdu, China; ^3^ Department of Pharmacy, Beijing Friendship Hospital, Capital Medical University, Beijing, China; ^4^ Beijing Key Laboratory of Bio-characteristic Profiling for Evaluation of Rational Drug Use, Beijing Shijitan Hospital, Capital Medical University, Beijing, China

**Keywords:** proteomics, glycoproteomics, N-glycopeptides, diabetes, complement

## Abstract

Diabetes has become a major public health concern worldwide, most of which are type 2 diabetes (T2D). The diagnosis of T2D is commonly based on plasma glucose levels, and there are no reliable clinical biomarkers available for early detection. Recent advances in proteome technologies offer new opportunity for the understanding of T2D; however, the underlying proteomic characteristics of T2D have not been thoroughly investigated yet. Here, using proteomic and glycoproteomic profiling, we provided a comprehensive landscape of molecular alterations in the fasting plasma of the 24 Chinese participants, including eight T2D patients, eight prediabetic (PDB) subjects, and eight healthy control (HC) individuals. Our analyses identified a diverse set of potential biomarkers that might enhance the efficiency and accuracy based on current existing biological indicators of (pre)diabetes. Through integrative omics analysis, we showed the capability of glycoproteomics as a complement to proteomics or metabolomics, to provide additional insights into the pathogenesis of (pre)diabetes. We have newly identified systemic site-specific N-glycosylation alterations underlying T2D patients in the complement activation pathways, including decreased levels of N-glycopeptides from C1s, MASP1, and CFP proteins, and increased levels of N-glycopeptides from C2, C4, C4BPA, C4BPB, and CFH. These alterations were not observed at proteomic levels, suggesting new opportunities for the diagnosis and treatment of this disease. Our results demonstrate a great potential role of glycoproteomics in understanding (pre)diabetes and present a new direction for diabetes research which deserves more attention.

## Introduction

Diabetes mellitus is a group of chronic diseases that could cause severe damage to various organs in human body, leading to disabling and life-threatening health complications (American Diabetes Association, 2019). It is estimated that 463 million individuals have diabetes mellitus globally in 2019, 90% of which are type 2 diabetes (T2D). By the year 2045, this number is projected to increase to 700 million (Saeedi et al., 2019). Due to its chronic nature, diabetes mellitus causes devastating personal suffering and socioeconomic costs (Hernández-Jiménez et al., 2019; Williams et al., 2020). Early detection may allow the early medical interventions and lifestyle modifications that can largely delay or even prevent the onset of diabetes and its complications (Federation, 2019). Therefore, new biomarkers that enhance early detection are in urgent need.

The routine clinical assessment of T2D is mainly based on the measurement of fasting plasma glucose (FPG), 2-h plasma glucose (2 h PG) from oral glucose tolerance test (OGTT), or glycosylated hemoglobin A1c (HbA1c) concentrations (the Association A.D., 2019). 2 h PG is a time-consuming and expensive test which is unpopular with both patients and physicians in recent years (Pippitt et al., 2016). Due to the great convenience of FPG measurement, FPG test has now been widely used for the detection of T2D, but with the limitations of lower sensitivity, repeatability, and reproducibility (Waugh et al., 2013; Gar et al., 2018). Compared with FPG and 2 h PG tests, HbA1c tests often detect T2D at later stages with less interpersonal variability when repeated. However, it is reported that many factors could influence the hemoglobin glycation independently of glycemia, including HIV treatment, race/ethnicity, pregnancy status, genetic background, and anemia/hemoglobinopathies (Kim et al., 2009; Eckhardt et al., 2012). Given the above limitations of current assessments and the strong demand for early medical intervention, current research efforts are sought to identify a serial of reliable, sensitive, and noninvasive biomarkers from human plasma to improve the accuracy of the early diagnosis for T2D patients and to help elucidate the underlying pathogenesis.

Recent advances in proteome technologies have now enabled the large-scale studies of proteins in tissues and body fluids, and identified many candidate protein biomarkers to diagnose T2D (Zhao et al., 2017; Nowak et al., 2018; Sohail et al., 2018; Jiang et al., 2019; Zhang et al., 2019; Zheng et al., 2020). However, few of those potential biomarkers have been successfully translated into clinical use. N-glycosylation is a heterogeneous posttranslational modification of proteins that plays a critical role in disease pathologies (Haltiwanger and Lowe, 2004; Ohtsubo and Marth, 2006; Vajaria and Patel, 2017; Pan et al., 2020). For example, we recently revealed that the alterations of N-glycopeptides were significantly associated with tumor progression in prostate and papillary thyroid carcinoma (Zhang et al., 2019). But until now, only a limited number of high-throughput glycoproteomic analyses have been carried out specifically for T2D (Miura and Endo, 2016; Sharma et al., 2020). Here, we propose that integrated proteomic and glycoproteomic analyses of human plasma will identify novel biomarkers that could provide additional insight into the pathogenesis of T2D and the assessments of which could be more accurate and sensitive for disease diagnosis than currently available test methods.

Therefore, we conducted comprehensive proteomic and glycoproteomic analyses in the plasma samples of 24 Chinese individuals, including eight T2D patients, eight prediabetic (PDB) subjects, and eight healthy control (HC) individuals. Our goal was to investigate the proteomic and glycoproteomic profile changes in the plasma of T2D patients, and also to provide new clues for exploring molecular and pathological mechanisms of T2D.

## Materials and Methods

### Sample Assembly

The study protocol was approved by the internal review boards and ethical committee boards of participating institutions. All patients have provided written informed consent. The study is registered at the Chinese Clinical Trial Registry (number: ChiCTR 1800014301).

Twenty-four plasma samples were collected for proteomic and glycoproteomic analyses, including eight T2D patients, eight PDB subjects, and eight HC individuals. All the patients were diagnosed as per the World Health Organization fasting blood glucose criteria (The World Health Organization, 2006).

### Sample Preparation

The 12 highest abundance proteins in plasma were first removed by the Pierce™ TOP 12 Abundant Protein Depletion Spin Columns (Thermo) according to the user manual. For proteomic analysis, the depleted samples were digested and prepared by the filter-aided sample preparation (FASP) method with optimization for plasma (Wiśniewski et al., 2009). In brief, 10 µl of plasma were depleted by the top 12 depletion kit, and then the depleted samples were diluted with UA buffer into 200 µl (8 M urea in 0.1 M Tris-HCl, pH 8.5) and centrifuged on a 30-kDa filter for 15 min. After centrifugation, 200 μl UA solution with 10 mM dithiothreitol (DTT) was added, and the reduction reaction was kept for 4 h at 37°C. The solution was removed by centrifugation at 14,000 *g*, and 200 µl UA solution with 50 mM iodoacetamide (IAA) was added. The mixture was incubated in the dark for 30 min at room temperature. The mixture was then washed three times with 200 μl UA and 200 μl ABC (50 mM ammonium bicarbonate) by centrifugation at 14,000 *g* for 15 min at room temperature. Then, 100 μl of ABC containing 0.1 μg/μl trypsin was added to each filter tube and incubated at 37°C for 12 h. The digested peptides were collected by washing the filter tubes with 100 μl water followed by 15 min of 14,000 *g* centrifugation twice. The peptide concentration was measured using a NanoDrop OneC (Thermo) at 280 nm absorbance (Jiang et al., 2019). The digested peptides were completely dried by SpeedVac centrifuge at 45°C (Eppendorf, Concentrator plus) and then stored at −80°C for further LC-MS/MS analysis.

For glycoproteomic analysis, 50 µl of 80% ACN/0.2% TFA solution was used to resuspend the digested peptides, and the concentration was determined by measuring 2 µl of suspension through NanoDrop OneC (Thermo) using the ε205 = 31 method. Meanwhile, Venusil HILIC (5 μm, 100 Å) was activated by washing with 0.1% TFA and 80% ACN/0.2%TFA three times for 10 min. 50 µg of each peptide suspension was mixed with 5 mg activated Venusil HILIC (5 μm, 100 Å) and subjected to a 2-h rotation at room temperature. Then, the mixtures were loaded onto a Pipet Tip (Axygen, Inc., Union City, CA, United States) packed with C8 membrane and washed twice with 80% ACN/0.2% TFA. Intact N-glycopeptides bound to HILIC column were collected by eluting with 70 μl of 0.1% TFA for three times. The eluents were then pooled and dried with SpeedVac centrifuge at 45°C (Eppendorf, Concentrator plus) and stored at −80°C for further LC-MS/MS analysis.

### LC-MS/MS Analysis

Samples were measured using LC-MS instrumentation consisting of an EASY-nLC 1,200 ultrahigh-pressure system (Thermo Fisher Scientific) coupled *via* a nano-electrospray ion source (Thermo Fisher Scientific) to an Orbitrap Fusion Lumos mass spectrometer (Thermo Fisher Scientific). For proteomic analysis, 0.5 μg of peptide mixture resolved in buffer A [0.1% formic acid (FA)] were loaded onto a 2-cm self-packed trap column (100-μm inner diameter, ReproSil-Pur C18-AQ, 3 μm; Dr. Maisch) using buffer A and separated on a 75-μm inner-diameter column with a length of 25 cm (ReproSil-Pur C18-AQ, 1.9 μm; Dr. Maisch) over a 120-min gradient (buffer A, 0.1% FA in water; buffer B, 0.1% FA in 80% ACN) at a flow rate of 600 nl/min (0–16 min, 3–10% B; 16–76 min, 10–22% B; 76–106 min, 22–30% B; 106–118 min, 30–90% B; 118–120 min, 90% B). The Orbitrap Fusion Lumos was set to the OT–OT mode. For a full mass spectrometry survey scan, the target value was 5 × 10^5^, and the scan ranged from 300 to 1,500 m/z at a resolution of 120,000 and a maximum injection time of 50 ms. For the MS2 scan, a duty cycle of 3 s was set with the top-speed mode. Only spectra with a charge state of 2–7 were selected for fragmentation by higher-energy collision dissociation with a normalized collision energy of 35%. The MS2 spectra were acquired in the Obitrap with an AGC target of 50,000 and a maximum injection time of 30 ms (Jiang et al., 2019).

For glycoproteomic analysis, 0.5 μg of N-glycopeptides reconstituted in 0.1% FA were separated over a gradient of 78 min at a flow rate of 400 nl/min (0–5 min, 6–10% B; 5–58 min, 10–22% B; 58–70 min, 22–50% B; 70–73 min, 50–90% B; 73–75 min, 90% B; 75–78 min, 90–10% B). For a full MS scan, the Orbitrap resolution was set to 120,000, with an AGC target value of 2 × 10^5^ for a scan range of 800–2,000 m/z and a maximum injection time of 100 ms. For MS2 scan, the higher-energy collision dissociation fragmentation was performed at the isolation width of 2 m/z and a 30% normalized collision energy. The MS2 spectra were collected in the Orbitrap detector with a 5 × 10^5^ AGC target, a maximum injection time of 250 ms, and a dynamic exclusion duration of 15 s (Zhang et al., 2019).

### Data Analysis

For proteomic analysis, the tandem mass spectra were searched against the human UniProt database (version 20200911, 20,375 sequences) using MaxQuant (version 1.6.12.0). Trypsin was selected as the proteolytic enzyme, and two missed cleavage sites were allowed. Cysteine carbamidomethylation was set as the fixed modification. The oxidation of M and acetylation of the protein N-terminal were set as the variable modifications. The first search mass tolerance was 20 ppm, and the main search peptide tolerance was 4.5 ppm. The false discovery rates of the peptide–spectrum matches (PSMs) and proteins were set to less than 1% (Cox and Mann, 2008; Jiang et al., 2019).

For glycoproteomic analysis, the raw MS files of enriched N-glycopeptides were searched against the human Swiss-Prot database (version 20200911, 20,375 sequences) with pGlyco v. 2.2.2 (Liu et al., 2017), as previously described by Zhang et al. (2019). The following parameters were used. Mass tolerances for the precursors and fragment ions were set as ±5 and ±20 ppm, respectively. Two missed cleavages sites were allowed for trypsin digestion. The fixed modification was carbamidomethylation of all cysteine residues (+57.02 Da). Variable modifications included oxidation of methionine (+15.99 Da), deamidation of asparagine (+0.98 Da), and acetylation of the protein N-terminal (+42.01 Da). The N-glycosylation sequon (N-X-S/T/C; X ≠ P) was modified by changing “N” to “J.” Both of these had the same mass. Quality control methods for intact glycopeptide identification were set to the 1% glycopeptide–spectrum matche (GPSM) false discovery rate (FDR). Quantification information (MS1 peak intensity) of N-glycopeptides spectra was acquired from the “allpeptide.txt” file in MaxQuant (Max Planck Gesellschaft, Munich, Germany) based on their unique MS/MS scan numbers from pGlyco 2.0 results.

### Bioinformatic Analysis

The bioinformatic analysis was mainly performed in the statistical analysis environment R (version 3.5.4) (The R Core Team, 2012). The expression matrix was normalized based on the median values of each sample and then subjected to quantitative analyses. In detail, the abundance of each protein or each N-glycopeptide was divided by the median value of the corresponding sample, respectively, and then subjected to log2 transformation for further analysis (Ge et al., 2018). Missing values were imputed using the minimum value across the proteome or glycoproteome data. Cellular localization and molecular function of identified proteins in proteomics and glycoproteomics were analyzed by using ClueGo app (v.2.3.3) plugin Cytoscape software (v.3.5.1), based on Gene Ontology (GO), Kyoto Encyclopedia of Genes and Genomes (KEGG), and Reactome data resources (Ashburner et al., 2000; Kanehisa et al., 2002; Shannon et al., 2003; Bindea et al., 2009; Croft et al., 2011). A simple linear model and moderated t-statistics were used to determine the significantly changed proteins and N-glycopeptides using R/Bioconductor package limma (Ritchie et al., 2015). The *p* values were corrected for multiple testing using the Benjamini–Hochberg (BH) procedure. In proteomics, proteins with a *p*-value < 0.05 and an absolute log2-fold change ≥1 were considered as differential expressed signatures. In glycoproteomics, the signature N-glycopeptides were determined based on a corrected *p*-value < 0.01 and an absolute log2-fold change ≥1. Functional characterization of identified signatures was performed by using the hypergeometric test (Falcon and Gentleman, 2008), based on three categories of GO cellular component (CC), molecular function (MF), and biological process (BP) gene sets obtained from the MSigDB database v.7.2 (Subramanian et al., 2005, http://software.broadinstitute.org/gsea/msigdb/index.jsp). All the interaction networks were constructed by using Cytoscape application.

### Data Availability Statement

The thermo .RAW files of proteomic and glycoproteomic datasets have been deposited to the ProteomeXchange Consortium *via* the iProX partner repository (Ma et al., 2019) with the dataset identifier PXD024105. Supplementary data are available online at http://bib.oxfordjournals.org/.

## Results

### Proteomic and Glycoproteomic Profiles of Plasma Samples

**FIGURE 1:**
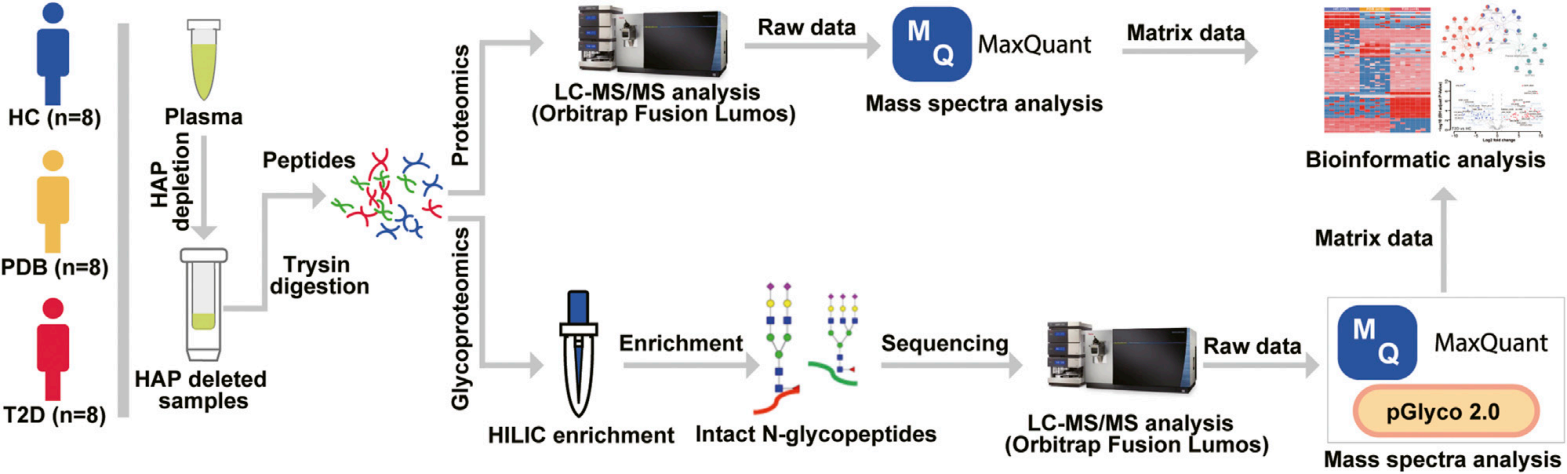
Mass spectrometry–based proteomic and glycoproteomic workflow. The 12 highest abundance proteins (HAPs) in the plasma were removed by Pierce™ TOP 12 Abundant Protein Depletion Spin Columns according to the user manual, and then the HAP depleted samples were digested by a modified FASP protocol. For proteomic analysis, the digested peptides were directly analyzed by an Orbitrap Fusion Lumos LC-MS/MS instrument, and mass spectra were searched against the human UniProt database using MaxQuant software before bioinformatic analysis. For glycoproteomic analysis, a Hilic enrichment analysis was conducted for digested peptides, and then the enriched N-glycopeptides were analyzed by LC-MS/MS instrument. The raw data were processed by MaxQuant and pGlyco software before bioinformatic analysis.

**FIGURE 2:**
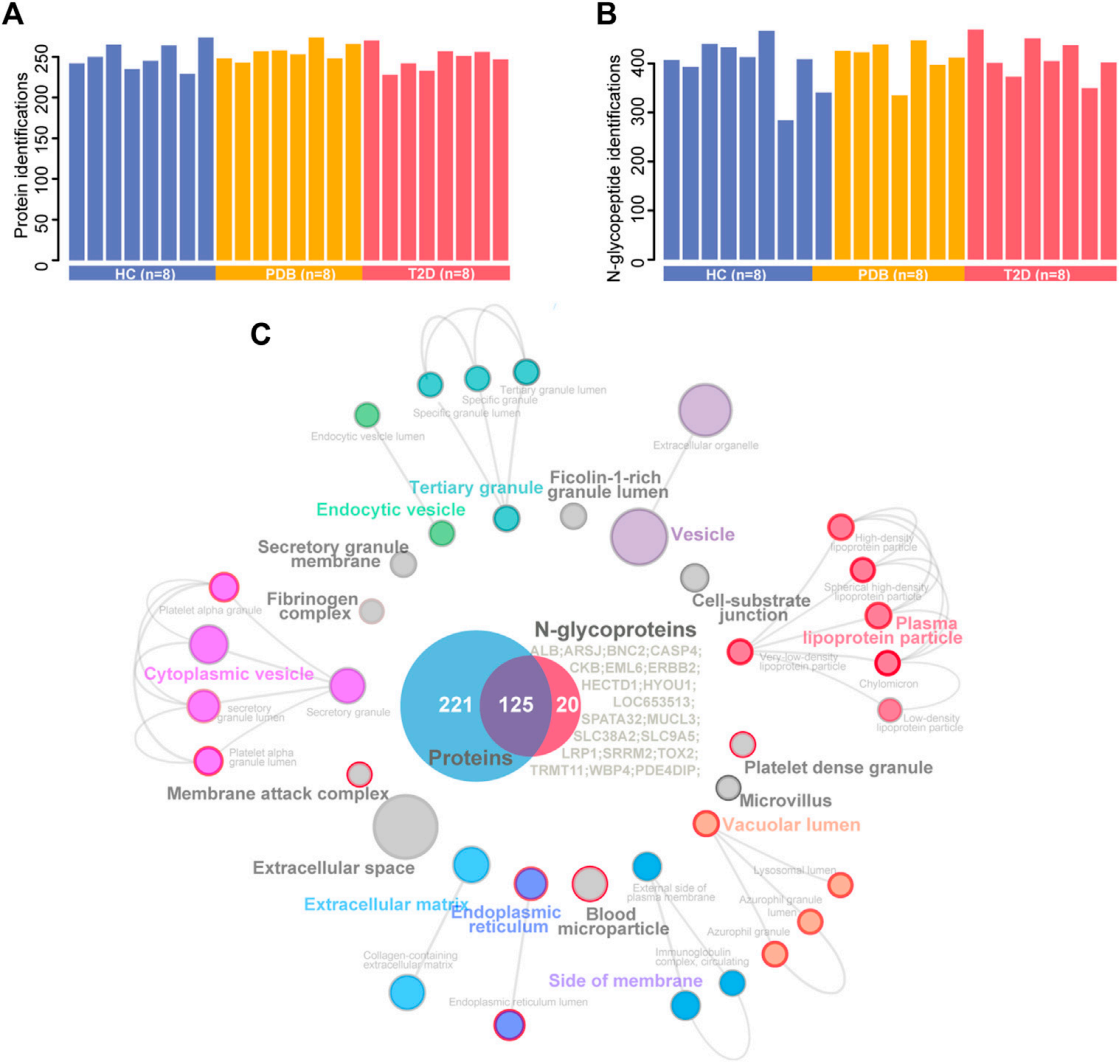
A summary of plasma proteomic and glycoproteomic analysis of our data. **(A, B)** Number of protein (**A**) and N-glycopeptide **(B)** identifications in HC (blue), PDB (yellow), and T2D (red) samples. **(C)** Cellular localization landscape of all proteins identified in proteomic and glycoproteomic analyses. A Venn diagram of proteins identified in proteomics or glycoproteomics is shown in the center of the picture, and the proteins exclusively identified in glycoproteomics are listed on the right of the Venn diagram. Cellular component interaction network of all proteins was constructed by the ClueGO app in Cytoscape software, based on Gene Ontology (GO) Cellular Component data resource. The component terms are connected based on the kappa score. The network modules are defined using the kappa score and annotated with different colors. For each module, the most significant pathway is highlighted by a colored name label. The size of the nodes indicates the number of identified proteins with this component. The components specifically enriched in glycoproteomics are labeled with a red node border.

We analyzed a panel of 24 plasma samples using a high-throughput integrated proteomic and glycoproteomic analysis protocol described in our recent study (Zhang et al. 2019; Figure 1 and *Methods*). The clinical information was provided in Supplementary Table S1. In total, we identified 346 proteins from the plasma samples that ranged from 228 to 274 proteins per individual, and identified 586 intact N-linked glycopeptides from 145 proteins, with an average of 406 N-glycopeptides per individual (Figures 2A,B). As shown in the Venn diagram, twenty proteins were exclusively identified in glycoproteomic profiles (Figure 2C).

To obtain a general overview of the cellular localizations and molecular functions of the identified proteins, the two protein groups were uploaded into ClueGo app separately as two clusters for pathway enrichment analysis and interaction network module analysis (Methods). For cellular localization analysis, a total of 39 component terms linked to nine network modules were enriched within all 366 proteins (Figure 2C, Supplementary Table S2). Most proteins were annotated as extracellular matrix, membrane attack complex, fibrinogen complex, lipoprotein particles, and blood microparticle components, or localized in the vesicle, lumen, and extracellular spaces. On the other hand, N-glycosylated proteins were preferentially found in the components of lipoprotein particle, membrane attack complex, platelet alpha granule, blood microparticle, endoplasmic reticulum, and lysosomal lumens, suggesting a specific cellular localization of N-glycosylated proteins.

**FIGURE 3:**
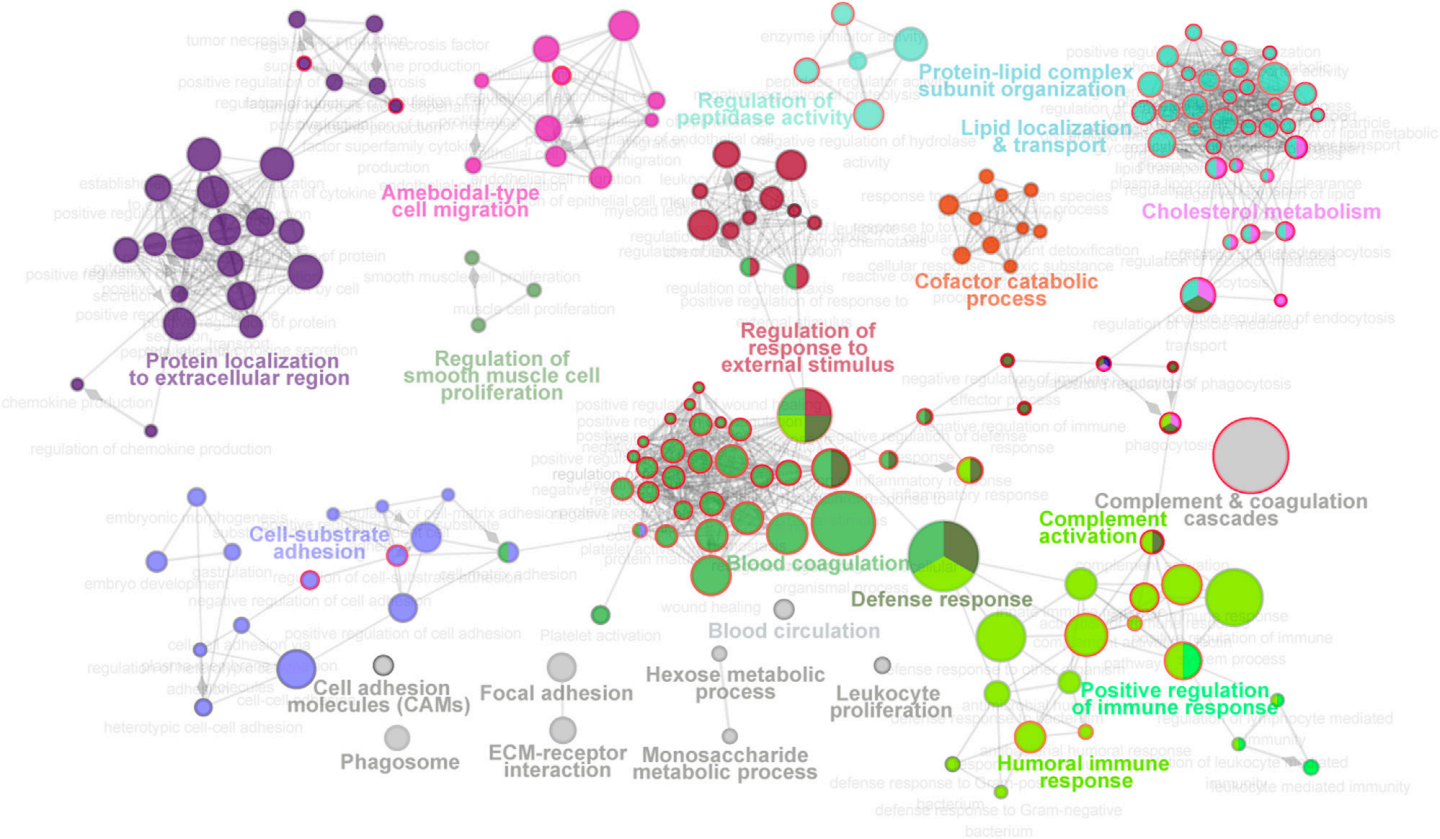
Molecular function landscape of all proteins identified in proteomics and glycoproteomics. Pathway interaction network of all proteins was constructed by the ClueGO app in Cytoscape software, based on Gene Ontology (GO), Kyoto Encyclopedia of Genes and Genomes (KEGG), and Reactome data resources. The pathways are connected based on the kappa score. The functional network modules are defined using the kappa score and annotated with different colors. For each module, the most significant pathway is highlighted by a colored name label. The size of the nodes indicates the number of identified proteins with this pathway. The pathways specifically enriched in glycoproteomics are labeled with a red node border.

For molecular function analysis, fourteen network modules related to 217 enriched pathways were notably identified within all the proteins, including pathways of plasma lipoprotein particle remodeling, humoral immune response, fibrinolysis, regulation of cholesterol transport, positive regulation of protein secretion, and cell substrate adhesion (Figure 3, Supplementary Table S2). However, N-glycosylated proteins were exclusively enriched in pathways of plasma lipoprotein particle remodeling, complement cascade, complement and coagulation cascades, binding and uptake of ligands by scavenger receptor, serine endopeptidase activity, platelet degranulation, and insulin growth factor (IGF) regulation, many of which are known to be associated with T2D (Fateh-Moghadam et al., 2005; Fadini et al., 2014; Haywood et al., 2019). The distinct aspects of the enrichment pathways suggested that glycoproteomic analysis can capture some new clues as a complementary to proteomic analysis, and highlighted that integrative of these two analyses will enable new advances in diabetes biology, diagnostics, and therapeutics.

### Signature Proteins and N-Glycopeptides for Type 2 Diabetic Patients

**FIGURE 4:**
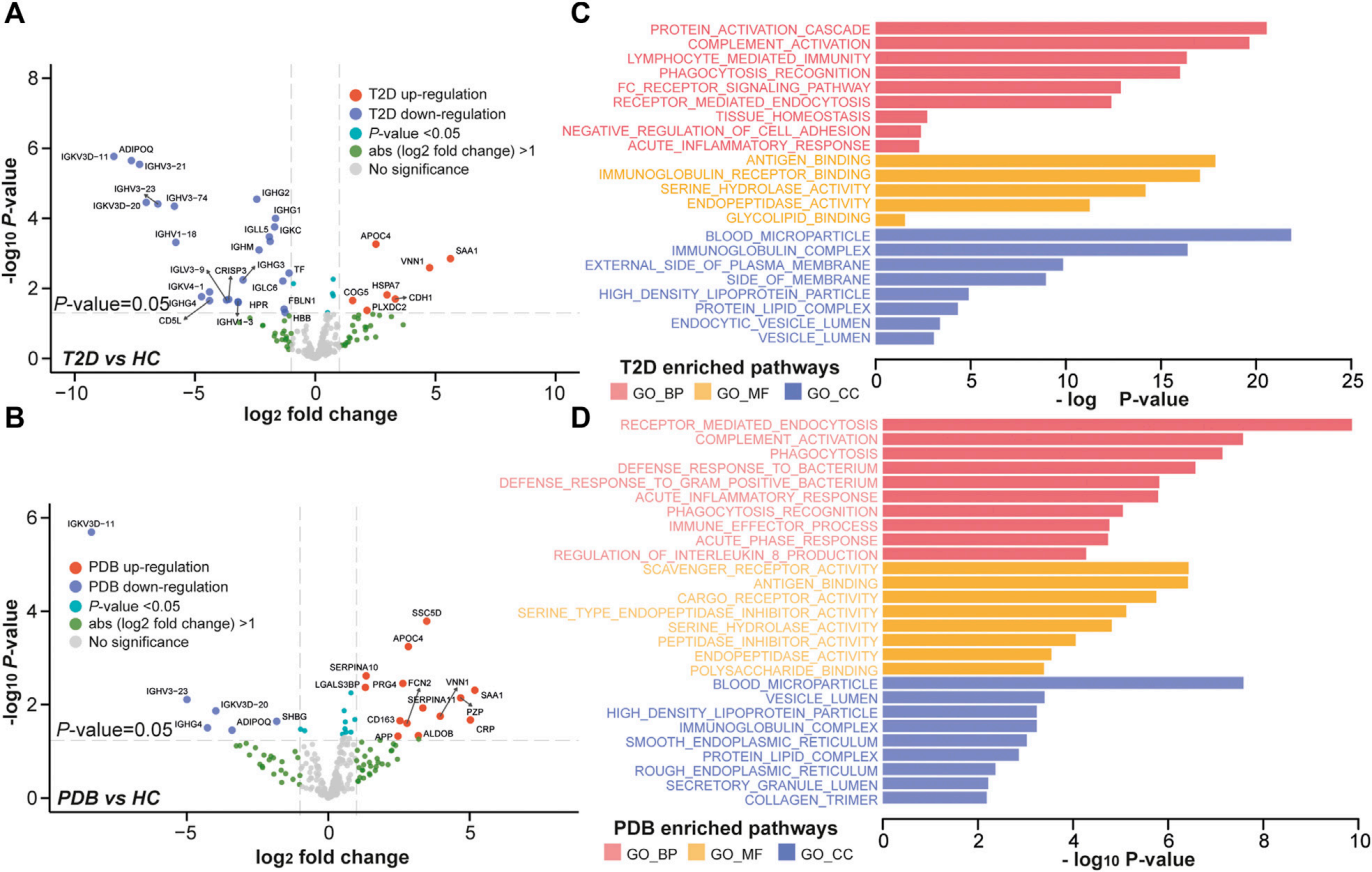
Proteomic alterations in T2D patients and PDB subjects. **(A, B)** Volcano plots of differential expressed proteins identified in T2D patients **(A)** or PDB subjects **(B)** compared to HC individuals. Red presents upregulation and blue indicates downregulation. Signature proteins were determined based on a *p*-value < 0.05 and an absolute log2-fold change ≥1. **(C, D)** GO enrichment results of the signature proteins identified in T2D **(C)** and PDB **(D)** groups.

To better understand the proteomic changes in the plasma of T2D patients, supervised pairwise comparisons were performed to identify signature proteins and N-glycopeptides using moderated t-statistics (Methods). Comparing with HC individuals, a total of 32 and 20 differential expressed proteins were identified in T2D and PDB groups, respectively, with an overlapping of eight proteins (*p*-value < 0.05 and absolute log2-fold change ≥1; Supplementary Table S3, Figures 4A,B). There were 19 proteins that distinguished T2D from PDB (Supplementary Table S3). Among these proteins, multiple previously described (pre)diabetic biomarkers were found, such as ADIPOQ, SAA1, TF, and VNN1, suggesting the robustness of our proteomic analysis (Memişoǧulları and Bakan, 2004; Siitonen et al., 2011; Anderberg et al., 2015; Kang et al., 2016). Gene ontology (GO) enrichment analysis was then performed to localize these signature proteins and determine their molecular functions and biological process. As shown in Figure 4C, the protein alterations of T2D occurred mainly in blood microparticle, immunoglobulin complex, the external side of the plasma membrane, high-density lipoprotein particle, protein–lipid complex endocytic vesicle lumen, and vesicle lumen. Their major molecular functions included antigen binding, immunoglobulin receptor binding, serine hydrolase activity, endopeptidase activity, and glycolipid binding. These protein alterations participated in protein activation cascade, complement activation, lymphocyte-mediated immunity, phagocytosis recognition, tissue homeostasis, negative regulation of cell adhesion, and acute inflammatory response processes, whereass the differential proteins in PDB were found mostly in blood microparticle, vesicle lumen, high-density lipoprotein particle, immunoglobulin complex, smooth endoplasmic reticulum, and protein–lipid complex (Figure 4D). Their main molecular functions were scavenger receptor activity, antigen binding, CAR receptor activity, serine-type endopeptidase inhibitor activity, and peptidase inhibitor activity. Differential proteins in PDB were involved in the receptor-mediated endocytosis, complement activation, and phagocytosis processes.

**FIGURE 5:**
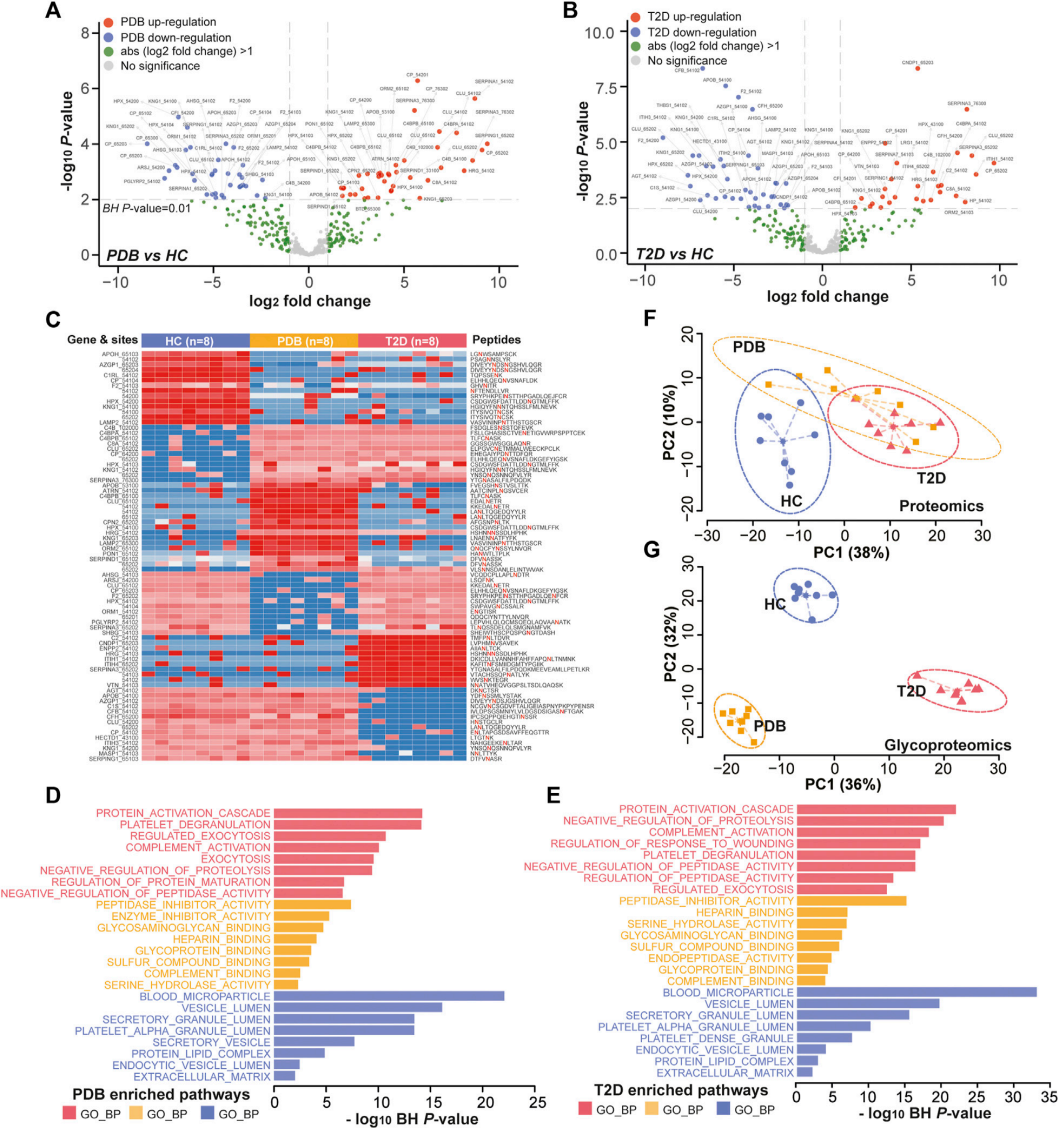
Glycoproteomic alterations in T2D patients and PDB subjects. **(A, B)** Volcano plots of differential expressed N-glycopeptides identified in PDB subjects **(A)** or T2D patients **(B)** or compared to HC individuals. Red presents upregulation and blue indicates downregulation. Signature proteins were determined based on a Benjamin–Hochberg corrected *p*-value < 0.01 and an absolute log2-fold change ≥1. **(C)** Heatmap depicting the relative abundance of signature N-glycopeptides. Samples are in columns and peptides are in rows. The gene names and glycan composition numbers are annotated on the left panel, and the peptide information is annotated on the right panel. **(D, E)** GO enrichment results of the mapped proteins of signature N-glycopeptides for PDB **(D)** and T2D **(E)** groups. **(F, G)** PCA plots of first two components of signature proteins **(F)** and N-glycopeptides **(G)**. The ellipse presents the 0.95 confidence intervals for each type.

In glycoproteomics, 75 N-glycopeptides from 40 proteins and 67 N-glycopeptides from 30 proteins were differentially expressed in PDB subjects and T2D patients as compared to HC individuals (BH *p*-value < 0.01 and absolute log2-fold change ≥1), with an overlapping of 25 N-glycopeptides from 14 proteins (Supplementary Table S4, Figures 5A,B). Using the same cutoff, 68 differential expressed N-glycopeptides from 45 proteins were identified between PDB and T2D groups (Supplementary Table S4). Among them, 78 N-glycopeptides from 42 proteins were specifically identified from one of the three groups, many of which had been reported as biomarkers for T1D or T2D at protein levels, but not at N-glycosylation levels yet, including AZGP1, CLU, C2, C4, F2, KNG1, and SERPING1 proteins (Zhang et al., 2013; Das and Kamalden, 2014; Figure 5C). Gene ontology (GO) enrichment analysis of the mapped proteins showed a high concordance with the cellular localizations and molecular functions of signature N-glycopeptides between PDB and T2D. As shown in Figures 5D,E, the alteration N-glycopeptides were mainly identified from the proteins that localized at blood microparticle, vesicle lumen, secretory granule lumen, platelet alpha granule lumen, and protein–lipid complex components, which had molecular functions of peptidase inhibitor activity, heparin binding, serine hydrolase activity, glycosaminoglycan binding, glycoprotein binding, and enzyme inhibitor activity, and involved in protein activation cascade, negative regulation of proteolysis, complement activation, and platelet degranulation processes.

Next, we performed unsupervised PCA analysis to investigate whether the signature panels can be served as indicators to predict T2D. As shown in Figure 5F, patients with T2D could be separated from HC individuals, but could hardly be separated from PDB subjects due to the overlapping at the protein level. However, a clear separation and a more tightened distribution of PCA plots were observed at N-glycoprotein levels (Figure 5G). In this view, glycoproteomic profiles and the signatures derived from them may be served as better predictive biomarkers for screening T2D patients. Depending on the connection of the pathogenesis of T2D development, these markers could be potential therapeutic targets in the future.

### Glycosylation Alterations of Complement Activation Pathways in Type 2 Diabetes

**FIGURE 6:**
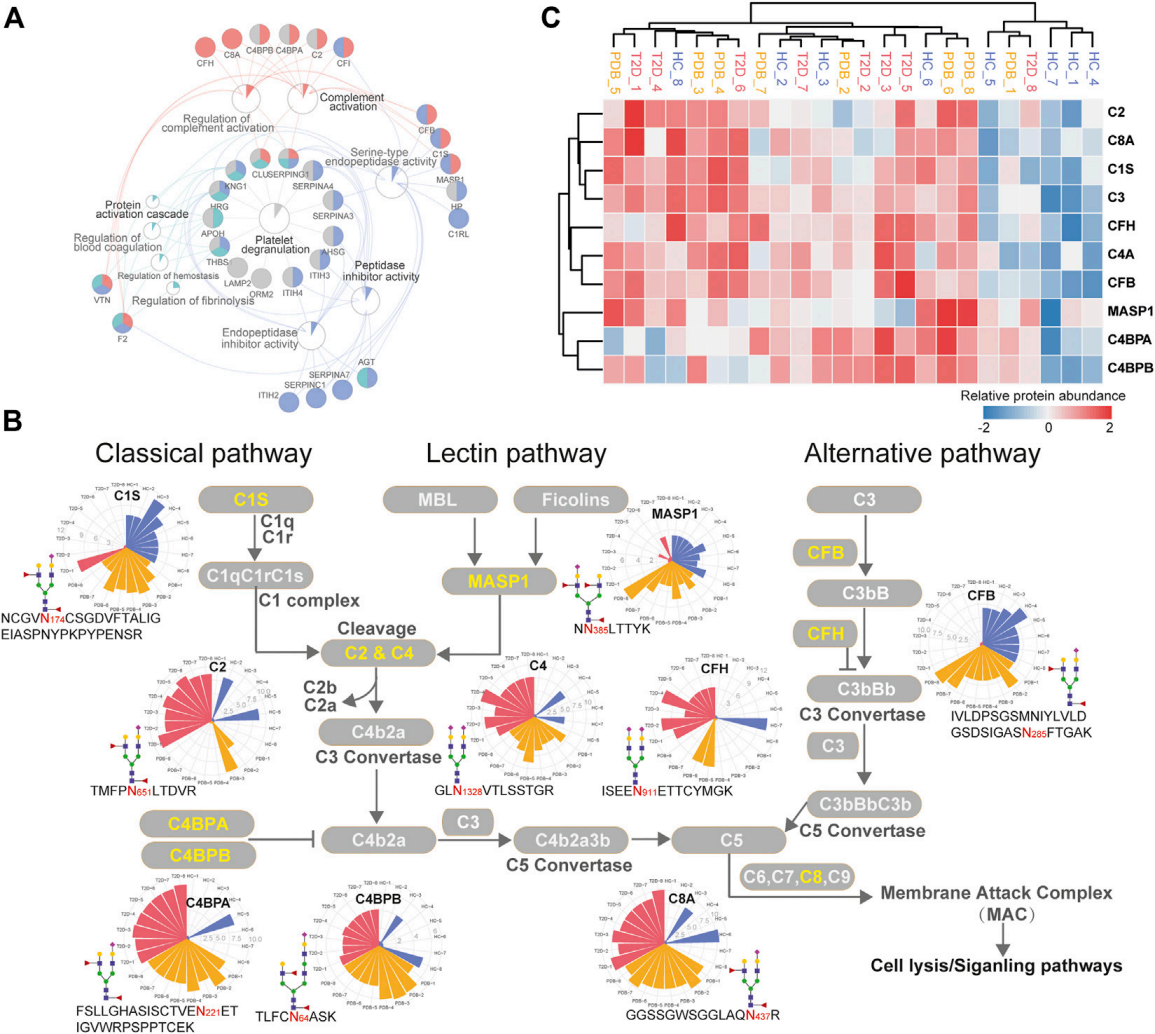
Dysregulation of N-linked glycosylation of complement activation pathways in T2D. **(A)** Pathway and protein interaction network of the mapped proteins from T2D signature N-glycopeptides. Distinct functional modules are annotated with different colors. The node size is determined by the number of proteins identified in that pathway, and the size of colored pie chart reflects the proportion of identified proteins of that pathway. **(B)** Network diagram summarizes relevant signature N-glycopeptides and signaling cascades involved in complement activation pathways. The proteins with N-glycosylation alterations are annotated as yellow characters, and their altered glycopeptides, glycosites, glycan compositions, and N-glycopeptide abundance are displayed. The relative abundance of N-glycopeptides is depicted as radar circles. Blue, HC individuals; yellow, PDB subjects; red, T2D patients. **(C)** Heatmap depicting the corresponding expression levels of the proteins discussed in complement activation pathways.

To further investigate molecular changes of T2D, the mapped proteins of differential N-glycopeptides identified from T2D *vs.* HC comparison were uploaded to ClueGo app in Cytoscape software for enriched pathway–protein interaction network module analysis. Four network modules were enriched, including the proteins involved in platelet degranulation, protein activation cascade, complement activation, and peptidase inhibitor activity (Figure 6A). In particular, a dysregulation of N-glycosylation of proteins involved in complement activation module were found in T2D patients. Normally, the first step to initiate complement system is the activation of three pathways, including classical pathways, lectin pathways, and alterative pathways (Flyvbjerg, 2017).

In classical and lectin pathways, C1 complex or the MASP1 protein recruits C2 and C4 to generate C4b2a C3 convertase, and in the alternative pathway, CFB binds to the spontaneous production of C3b to form C3bBb C3 convertase, facilitating the cleavage of C3 to generate C4b2a3b or C3bBbC3b C5 convertase and then activating the complement system through membrane attack complex (MAC) proteins (Chen et al., 2014; Ghosh et al., 2015). As shown in Figure 6B, the N-glycopeptides NCGV**N**_**174**_CSGDVFTALIGEIASPNYPKPYPENSR-HexNAc4Hex5-NeuAc1Fuc2 from the C1s protein, N**N**_**385**_LTTYK-HexNAc4Hex5NeuAc2Fuc1 from the MASP1 protein, and IVLDPSGSMNIYLVLDGSDSIGAS**N**_**285**_FTGAK-HexNAc4Hex5NeuAc1Fuc2 from the CFB protein, all of which modified by fucosylated glycans, were clearly down-regulated in the plasma of T2D patients. By contrast, the N-glycopeptides TMFP**N**_**651**_LTDVR-HexNAc4Hex5NeuAc1Fuc2–carrying fucosylated glycans from the C2 protein and GL**N**_**1328**_VTLSSTGR-HexNAc4Hex5NeuAc2 from the C4 protein were significantly up-regulated in T2D patients, which are downstream targets of C1 complex and the MASP1 protein, as well as TVLTPATNHMG**N**_**85**_VTFTIPANR-HexNAc2Hex7 from the C3 protein, which is the downstream target of C3 convertase. In addition, the N-glycopeptides that were derived from C3 convertase inhibitors were of higher abundance in T2D patients than in HC individuals. These included FSLLGHASISCTVE**N**_**221**_ETIGVWRPSPPTCEK-HexNAc4Hex5NeuAc1Fuc2–carrying fucosylated glycans, TLFC**N**_**64**_ASK-HexNAc5Hex6NeuAc1Fuc2–carrying fucosylated glycans, and KAFIT**N**_**81**_FSMIIDGMTYPGIIK-HexNAc4Hex5NeuAc2 from C4BPA, C4BPB, and CFH proteins, respectively. Suppose that N-glycopeptides abundance is positively associated with molecular function, our results above suggested that the complement activation pathways were inhibited in T2D from the perspective of glycoproteomics, and *vice versa*. Further studies will be needed to assess the biological relevance of the findings.

## Discussion

Diabetes is a global health problem that affects more than 400 million peoples each year (Saeedi et al., 2019). The routine clinical tests for the diagnosis and follow-up of diabetes have certain limitations; for example, they may not be sufficiently sensitive at early disease stages (the Association A.D., 2019). Recent advances in proteomics and glycoproteomics technologies hold great potential in providing additional insights into the disease biology and discovering new biomarkers (Vajaria and Patel, 2017; Zheng et al., 2020). Herein, we presented the global proteomic and glycoproteomic landscape in the fasting plasma of the 24 Chinese participants, and provided some clues for diagnosis and treatment of T2D.

Our integrated analysis suggested a specific cellular localization and molecular function of N-glycosylated proteins and highlighted the fundamental discrepancy between proteomic and glycoproteomic profiles. In proteomics, a total of 32 and 20 differential expressed proteins were identified in T2D and PDB groups compared to the HC group, which were mainly localized in blood microparticle, immunoglobulin complex, protein–lipid complex, and vesicle lumen components, and involved in antigen binding, immunoglobulin receptor binding, protein activation cascade, complement activation, receptor-mediated endocytosis, and phagocytosis pathways. Within our selected markers, there are actually several proteins that have already been reported in previous studies, such as ADIPOQ, SAA1, TF, and VNN1 proteins. For instance, VNN1 is a pantetheinase that plays a crucial role in gluconeogenesis and lipogenesis, affecting multiple metabolic pathways (Chen et al., 2014; Kang et al., 2016). Clinical investigations showed that the level of VNN1 was increased in the blood of diabetic patients (Chen et al., 2014). Our results further supported the involvement of VNN1 in the pathological process of diabetes, thus making VNN1 as an important biomarker candidate for the diagnosis of this disease. Moreover, our results agreed with previous findings that the expression of TF and ADIPOQ was decreased and the expression of SAA1 was increased in the plasma of T2D patients, which enhanced the robustness of our proteomic findings and highlighted the potential of our signature proteins serving as biomarkers for the early detection of (pre)diabetes (Memişoǧulları and Bakan, 2004; Siitonen et al., 2011; Anderberg et al., 2015).

In glycoproteomics, 75 and 67 differential expressed N-glycopeptides were identified from 40 proteins in PDB subjects and 30 proteins in T2D patients, respectively. The mapped proteins of these N-glycopeptides are known to be involved in platelet degranulation, protein activation cascade, peptidase inhibitor activity, and complement activation, some of which have been identified as potential biomarkers for T1D or T2D, including AZGP1, CLU, C2, C4, F2, KNG1, and SERPING1 (Zhang et al., 2013; Das and Kamalden, 2014). In addition, some family members of SERPING1, including SERPINA3, SERPINA4, SERPINA7, and SERPINC1, were also found to be dysregulated in T2D patients through glycoproteomics in our study, which were involved in platelet degranulation and peptidase inhibitor pathways (Figure 6A). Within their gene families SERPINA6, SERPINB2, and SERPINB8 have long been known as biomarkers for diabetes (Wang et al., 2008; Zhang et al., 2013). However, all the biomarkers that were reported previously were identified from the proteomic level, and the changes of their N-glycosylation are first time reported in the plasma of T2D patients by our study. The potential role of these N-glycosylation changes and their contribution to the development of T2D warrant further mechanistic investigation.

The major glycoproteomic changes of T2D patients were characterized by the dysregulation of N-glycopeptides involved in the activation of complement pathways. The complement system plays an important role in innate immune defense and humoral immunity, which is typically thought to be activated through classical, lectin, and alternative pathways, and increasing evidence suggests their pathological roles in the development of diabetes and its complications (Acosta et al., 2000; Yang et al., 2013). The classical pathway is activated by binding of the C1 complex (comprising C1q, C1r, and C1s) to antibodies, while the lectin pathway is activated by MBL-associated serine proteases (MASP1 or MASP2) after recognition of carbohydrates by mannose-binding lectin (MBL) or recognition of N-acetylglucosamine residues by ficolins, facilitating the cleavage of C2 and C4 to form C4b2b C3 convertase (Ghosh et al., 2015; Flyvbjerg, 2017). The alternative pathway is triggered through the spontaneous activation of C3, and the produced C3b forms C3bBb C3 convertase under the action of complement factor B (CFB) and complement factor D (CFD). All these pathways lead to the cleavage of C3 and converge into one pathway, eventually resulting in the formation of membrane attack complex (MAC), the main effector of complement-mediated tissue damage, which lyses, damages, or activates target cells (Ghosh et al., 2015; Flyvbjerg, 2017).

In the present study, we found that several N-glycopeptides from C1s, MASP1, and CFP proteins were clearly down-regulated in T2D patients, while N-glycopeptides from C2, C4, C4BPA, C4PBB, and CFH proteins were inversely up-regulated. According to these results, we speculate that at glycoproteomic levels, the decreased N-glycopeptides of positive regulator proteins C1s and MASP1 may hinder the cleavage of C2 and C4 to generate C4b2a C3 convertase, and the decreased N-glycopeptide of CFB may inhibit the formation of C3Bb C3 convertase, resulting in increased N-glycopeptide levels of C2, C4, and C3 proteins, which might suppress the complement activation system in one way. Meanwhile, the increased levels of the C3 convertase inhibitors C4BPA, C4BPB, and CFH would result in the failure of C3 cleavage and further suppress the complement activation system (Figure 6B). However, these alterations were not observed at proteomic levels (Figure 6C). Moreover, many immunoglobulin biomarkers associated with the complement activation pathway are found to be lost in T2D patients instantly, suggesting a suppressing status of complement system. In specific, we found that most N-glycosylation alterations occurred in the complement pathway are modified with fucosylated glycans, indicating a potential role of this glycan modification in the development of diabetes. Overall, these results suggest that N-glycosylation dysregulation in complement activation pathways may be closely associated with the pathogenesis of T2D, thereby requiring further analyzing and exploring in future studies.

In addition to the complement system we focused on, we also observed other interesting N-glycosylation changes in other pathways. For instance, zinc-alpha-2-glycoprotein (ZAG) is a soluble protein that can be identified in plasma. As an adipokine, ZAG is mainly secreted by subcutaneous and visceral adipocytes and is involved in stimulating adipocytes to induce lipid degradation (Yang et al., 2013). It has been reported that ZAG is related to the markers of insulin resistance or obesity and may play a role in the pathogenesis of insulin resistance (IR) (Bouchara et al., 2018). ZAG index can be served as an indicator to identify IR (Qu et al., 2016). The alteration of intact N-glycopeptide may play a vital role in the development of T2D and can be used as a potential indicator. Ceruloplasmin (Cp), another one worth mentioning, is a copper-binding glycoprotein with ferroxidase activity. As a key protein participated in iron metabolism and antioxidant defense, the level of Cp during the acute phase is regulated in response to inflammation, infection, copper and iron metabolism disorders, and vascular injury (Daimon et al., 1998; Golizeh et al., 2017). It has been found that the increase in the plasma Cp level in diabetic patients may be a signal of increased oxidative stress (Cunningham et al., 1995). The progression of T2D is associated with oxidative stress, iron metabolism disorders, and inflammation (Golizeh et al., 2017). Therefore, the change of the Cp N-glycopeptide level may also predict the occurrence and development of diabetes. Briefly, the alternation of N-glycopeptide prior to protein change may help us diagnose diseases in advance.

In summary, this work provides a comprehensive plasma proteomic and glycoproteomic resource for pathogenic investigation of diabetes and identifies a set of predictive biomarkers that might enhance the efficiency and accuracy based on current existing biological indicators of (pre)diabetes. To our knowledge, the systemic dysregulation of the glycoproteomic level in the complement activation system is first time identified to be a characteristic of T2D, which sheds new light on the pathogenesis of this disease and suggests new possibilities for diagnosis and treatment. As we see it, although the number of samples presented here is too few to make robust conclusions, this work illustrates the capability of glycoproteomics which serves as complement to proteomics or metabolomics and could provide additional detailed insights into pathways and processes that drive the development of diabetes disease. It thereby presents a new direction for understanding diabetic pathogenesis and deserves more attention.

## Data Availability

The datasets presented in this study can be found in online repositories. The names of the repository/repositories and accession number(s) can be found in the article/Supplementary Material.
